# Awareness and utilization of antenatal care services: A comparative study between educated and uneducated mothers

**DOI:** 10.6026/973206300223077

**Published:** 2026-05-31

**Authors:** Kiranmai Pandi, Balasubramanian R, Anshuli Trivedi, Chaitra C.M, Aaliya Kazi, Neha M Mahajan

**Affiliations:** 1 Department of Community Medicine, Gandhi Medical College, Bhopal, Madhya Pradesh, India; 2 Department of Obstetrics and Gynaecology, Gandhi Medical College, Bhopal, Madhya Pradesh, India; 3 Department of Community and Family Medicine, All India Institute of Medical Sciences, Bhopal, Madhya Pradesh, India; 4 Department of Obstetrics and Gynaecology, Indira Gandhi Institute of Medical Sciences, Patna, Bihar, India; 5 Department of Obstetrics and Gynaecology, Max Hospital, Gurugram, Haryana, India

**Keywords:** Antenatal care (ANC), awareness, utilization, maternal education, pregnancy, public health

## Abstract

Differences in awareness and utilization of antenatal care services between educated and uneducated mothers remain insufficiently explored. Therefore, it is of interest to compare the level of awareness and use of antenatal care services among 120 mothers attending a tertiary care center. Data were collected using a pre-tested structured questionnaire assessing awareness and utilization, with analysis performed using SPSS and chi-square test. Educated mothers demonstrated significantly higher awareness and better utilization of antenatal care services compared to uneducated mothers (p < 0.05). Thus, we show the critical role of maternal education in improving antenatal care uptake, supporting targeted interventions to enhance maternal and fetal outcomes.

## Background:

Maternal health is a significant global priority in the field of public health, especially in developing nations where the preventable maternal and neonatal morbidity and mortality still represent a challenge. One of the most important interventions to effectively monitor the pregnancy, identify complications at the earliest stages and offer preventive and promotional healthcare services to pregnant mothers is the antenatal care (ANC) [1]. Quality ANC has also been demonstrated to greatly decrease negative pregnancy outcomes and boost maternal and neonatal survival [2]. The World Health Organization suggests at least eight antenatal contacts during pregnancy in order to receive all-inclusive care, such as early identification of complications, nutritional supplementation, immunization and counseling [3]. In most of the low- and middle-income nations, such as India, however, the use of ANC services is still less than optimal and disparities are still significant among the various population groups [4]. It has been established that education is an important determinant of health-seeking behavior. Educated women would be more apt to realize the significance of antenatal care, how to be cautious during pregnancy and receive healthcare services in a timely fashion [5]. Conversely, lack of awareness, cultural beliefs, financial constraint and limited autonomy are some of the barriers that require uneducated mothers to use ANC services [6]. In India, even with the introduction of national programs, there are gaps in effective delivery and utilization of maternal healthcare services [7]. Research has indicated that institutional births are on the rise, but the quality and suitability of antenatal care is uneven, especially among the socioeconomically disadvantaged and the less educated people [8]. ANC awareness entails an understanding of such fundamental elements as early pregnancy registration, the number of visits, iron and folic acid supplement, tetanus vaccine and identification of warning signs of the condition, including bleeding, an intense headache and fetal lack of movement [9]. Utilization, in its turn, is defined as the actual usage of such services which depends on various factors, such as education, accessibility, cultural practices and healthcare infrastructure [10]. The past literature has clearly shown that maternal education is positively correlated with the use of antenatal services. Well-educated mothers will be more inclined to make the recommended number of ANC visits, use the supplements and obtain skilled care during pregnancy and childbirth [11]. On the other hand, lack of education among mothers leads to a tendency of those mothers to underuse the services provided, which makes them more likely to develop preventable complications [12]. Therefore, it is of interest to evaluate and compare the level of awareness and use of antenatal care services between the educated and uneducated mothers, as well as to identify the gaps and propose the strategies to enhance the level of maternal healthcare services utilization.

## Materials and Methodology:

## Study design:

This study was conducted as a comparative cross-sectional study aimed at assessing and comparing awareness and utilization of antenatal care (ANC) services among educated and uneducated mothers.

## Study setting:

The study was carried out in the Department of Obstetrics and Gynecology of a tertiary care teaching hospital in India, including both antenatal clinic (ANC) and postnatal wards.

## Study duration:

The study was conducted over a period of 6 months (e.g., January 2025 to June 2025).

## Sample size:

A total of 120 mothers were included in the study.

[1] Group I (Educated mothers): 60

[2] Group II (Uneducated mothers): 60

## Operational definition:

[1] Educated mothers: Women who had completed at least primary school education

[2] Uneducated mothers: Women with no formal schooling

## Sampling technique:

A consecutive sampling method was used, where eligible participants attending the hospital during the study period were recruited until the desired sample size was achieved.

## Inclusion criteria:

[1] Pregnant women (in any trimester) or postnatal mothers (within 6 weeks of delivery)

[2] Women willing to participate in the study

[3] Women with complete antenatal records (if available)

## Exclusion criteria:

[1] Women with severe illness or complications affecting communication

[2] Women unwilling to give consent

[3] Incomplete or unreliable responses

## Data collection tool:

Data were collected using a pretested, structured questionnaire, designed in the local language and English.

The questionnaire consisted of three sections:

## Section A:

## Socio-demographic details:

[1] Age

[2] Education level

[3] Occupation

[4] Socioeconomic status

[5] Parity

## Section B:

## Awareness of antenatal care:

Knowledge regarding:

[1] Ideal time of ANC registration

[2] Recommended number of ANC visits

[3] Importance of iron and folic acid supplementation

[4] Tetanus toxoid immunization

[5] Nutritional requirements during pregnancy

[6] Recognition of danger signs (bleeding, swelling, headache, decreased fetal movements)

## Section C:

## Utilization of antenatal care services:

[1] Timing of first ANC visit

[2] Number of ANC visits completed

[3] Compliance with iron-folic acid tablets

[4] Immunization status

[5] Laboratory investigations performed

[6] Institutional delivery planning

## Scoring system:

[1] Awareness score:

1) Each correct response = 1 point

2) Total score categorized as:

a) Poor awareness

b) Moderate awareness

c) Good awareness

[2] Utilization assessment:

1) Adequate utilization defined as:

a) Early registration (within first trimester)

b) ≥4 ANC visits

c) Consumption of ≥100 iron-folic acid tablets

d) Complete tetanus immunization

## Data collection procedure:

[1] Participants were interviewed face-to-face after obtaining informed consent

[2] Confidentiality was maintained

[3] Medical records were reviewed (where available) to validate responses

## Outcome measures:

## Primary outcomes:

[1] Level of awareness regarding antenatal care

[2] Utilization of antenatal care services

## Secondary outcomes:

[1] Association between maternal education and ANC utilization

[2] Identification of gaps in awareness and service uptake

## Statistical analysis:

Data were entered into Microsoft Excel and analyzed using SPSS software (version 26.0, IBM Corp., Armonk, NY, USA).

[1] Descriptive statistics:

1) Mean, standard deviation

2) Frequencies and percentages

[2] Inferential statistics:

1) Chi-square test for comparison between groups

2) Independent t-test (if applicable for scores)

3) p-value < 0.05 was considered statistically significant

## Results:

A total of 120 mothers were included in the study, divided into educated mothers: 60 and uneducated mothers: 60. Educated mothers showed significantly higher awareness across all parameters. Awareness of early registration was twice as high (80% vs 40%). Knowledge of danger signs was 109% higher in educated mothers. Overall awareness difference was statistically highly significant (p < 0.001) (Table 1). Utilization of antenatal care services was higher among educated mothers than uneducated mothers. Early ANC registration was observed in 42 (70%) educated mothers compared with 25 (41.7%) uneducated mothers. Similarly, at least four ANC visits were completed by 46 (76.7%) educated mothers versus 28 (46.7%) uneducated mothers, and consumption of at least 100 IFA tablets was reported by 40 (66.7%) educated mothers compared with 22 (36.7%) uneducated mothers. Complete TT immunization was also higher among educated mothers, with 55 (91.7%) compared with 45 (75%) in uneducated mothers. The overall difference was statistically significant (p = 0.002) (Table 2). Good awareness was more than double in educated mothers (66.7% vs 30%). Poor awareness was 4 times higher in uneducated mothers. Adequate utilization was 69% higher among educated mothers. Overall differences were highly significant (p < 0.001) (Table 3).

## Discussion:

The current research indicates a considerable relationship between maternal education and level of awareness as well as the use of antenatal care services (ANC). Mothers who were educated had a better knowledge and were in better practice of utilization than the uneducated mothers. The results are consistent with national surveys that are conducted in large numbers that suggest maternal education to be a major determinant of ANC utilization. The National Family Health Survey data have been analyzed to reveal that less educated women are much less likely to receive the complete antenatal care [1, 13]. In the current study, the levels of awareness of the key ANC elements of early registration, recommended visits and danger signs were significantly higher among educated mothers. The same results were reported in a research done in Delhi, where the knowledge about ANC was highly linked with education level (p < 0.001) and had a direct effect on the use of the service [2]. The percentage of mother who attended 4 or more ANC visits was more in educated mothers (76.7% versus 46.7%). This is in line with past research that has shown that education increases health-seeking behavior through the effect of improving knowledge about the benefits of attending to the antenatal care and also through increased contact with the healthcare systems [14]. Iron-folic acid (IFA) compliance was also significantly higher among educated mothers. According to the national statistics, approximately 0% of women use IFA during the recommended time, which identifies some gaps in adherence, especially in less educated groups [1]. Better compliance in the educated population of mothers in this study is due to increased awareness and compliance to medical advice. Another finding of the current study was that knowledge regarding danger signs was considerably less among the uneducated mothers and this might contribute to the delay in seeking healthcare and the risk of developing complications. These results are also reported to coincide with community based studies in India where maternal morbidity was found to be higher due to poor awareness [4]. The ANC utilization has been well documented to be affected by seasonal and socio-demographic factors. Education, socio-economic status and access to healthcare facilities are some of the factors that are vital in determining the patterns of ANC use [5]. The existing results support the role of education as a key determinant. Increased use of ANC services by educated mothers could also be attributed to increased exposure to mass media, autonomy in decision making and easy access to information on healthcare. There is evidence that women are more likely to be involved in health systems and use preventive services when they are better educated [6]. Although there have been improvements in maternal health programs, there still exists disparities. It has been revealed that in India, relatively, only a fifth of women access quality ANC services, which means that there is a gap in awareness as well as the delivery of these services [7, 15]. These differences are also put into perspective at the individual-level in the findings of the present study. The paper highlights the importance of special health education programs to reach uneducated mothers. The gap should be closed by community-based programs, frontline health workers and enhancing maternal health.

## Conclusion:

Educated mothers had been found to be much more aware and used antenatal care services than uneducated mothers. Maternal education had a strong relationship with better health-seeking behavior and adherence to ANC recommendations. There are still considerable disparities between uneducated mothers, especially in terms of knowledge of danger signs and access to the necessary services.

## Figures and Tables

**Table 1:** Awareness of antenatal care components

**Parameter**	**Educated (n=60)**	**Uneducated (n=60)**	**p-value**
Knowledge of early registration	48 (80%)	24 (40%)	
Knowledge of ≥4 ANC visits	45 (75%)	20 (33.3%)	
Awareness of IFA supplementation	50 (83.3%)	28 (46.7%)	
Knowledge of TT immunization	52 (86.7%)	35 (58.3%)	
Awareness of danger signs	46 (76.7%)	22 (36.7%)	<0.001

**Table 2:** Utilization of antenatal care services

**Parameter**	**Educated (n=60)**	**Uneducated (n=60)**	**p-value**
Early ANC registration	42 (70%)	25 (41.7%)	
≥4 ANC visits	46 (76.7%)	28 (46.7%)	
≥100 IFA tablets consumed	40 (66.7%)	22 (36.7%)	
Complete TT immunization	55 (91.7%)	45 (75%)	0.002

**Table 3:** Overall awareness and utilization levels

**Category**	**Educated (n=60)**	**Uneducated (n=60)**	**p-value**
Good awareness	40 (66.7%)	18 (30%)	
Moderate awareness	15 (25%)	22 (36.7%)	
Poor awareness	5 (8.3%)	20 (33.3%)	
Adequate utilization	44 (73.3%)	26 (43.3%)	<0.001
